# Microfabricated Physical Spatial Gradients for Investigating Cell Migration and Invasion Dynamics

**DOI:** 10.1371/journal.pone.0020825

**Published:** 2011-06-10

**Authors:** Michael Mak, Cynthia A. Reinhart-King, David Erickson

**Affiliations:** 1 Department of Biomedical Engineering, Cornell University, Ithaca, New York, United States of America; 2 Sibley School of Mechanical and Aerospace Engineering, Cornell University, Ithaca, New York, United States of America; The University of Akron, United States of America

## Abstract

We devise a novel assay that introduces micro-architectures into highly confining microchannels to probe the decision making processes of migrating cells. The conditions are meant to mimic the tight spaces in the physiological environment that cancer cells encounter during metastasis within the matrix dense stroma and during intravasation and extravasation through the vascular wall. In this study we use the assay to investigate the relative probabilities of a cell 1) permeating and 2) repolarizing (turning around) when it migrates into a spatially confining region. We observe the existence of both states even within a single cell line, indicating phenotypic heterogeneity in cell migration invasiveness and persistence. We also show that varying the spatial gradient of the taper can induce behavioral changes in cells, and different cell types respond differently to spatial changes. Particularly, for bovine aortic endothelial cells (BAECs), higher spatial gradients induce more cells to permeate (60%) than lower gradients (12%). Furthermore, highly metastatic breast cancer cells (MDA-MB-231) demonstrate a more invasive and permeative nature (87%) than non-metastatic breast epithelial cells (MCF-10A) (25%). We examine the migration dynamics of cells in the tapered region and derive characteristic constants that quantify this transition process. Our data indicate that cell response to physical spatial gradients is both cell-type specific and heterogeneous within a cell population, analogous to the behaviors reported to occur during tumor progression. Incorporation of micro-architectures in confined channels enables the probing of migration behaviors specific to defined geometries that mimic *in vivo* microenvironments.

## Introduction

Metastasis is the leading cause of cancer related deaths. The mechanisms and effects of metastasis often span large spatial and temporal scales, which make any experimental and analytical characterization difficult. To address the need for characterizing and screening the metastatic potential of cells, researchers have begun looking for mechanical markers at the single cell level [1]–[3]. This is particularly useful since metastasis has been characterized as an inefficient process that eventually works due to very small subpopulations of successfully invasive cells. This notable feature has also led to an emphasis on understanding the importance of heterogeneity within cancer cell populations, as certain subpopulations are speculated to be more apt to progress through the entire metastatic cascade. The importance of heterogeneity and the implications of different heterogeneous phenotypes on cancer metastasis, however, have not yet been fully resolved [4]–[9]. Of particular interest are phenotypes that promote motility and invasiveness, as these properties are often associated with metastasis.

**Figure 1:**
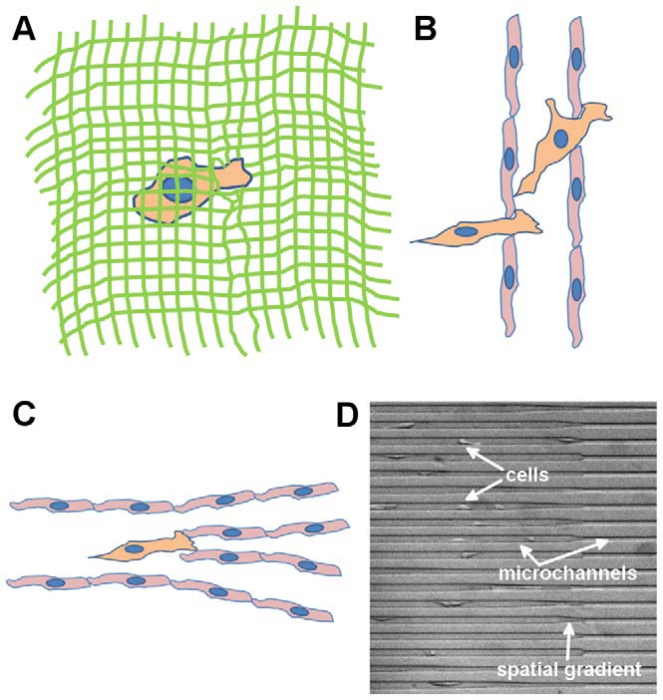
Cells encountering spatial gradients in physiological and simulated environments. **A–C.** A cancer cell (orange) is (A) navigating through small pores in the extracellular matrix (green) as it is invading through the tumor stroma, (B) squeezing through the endothelium (red cells) during intra- and extravasation, and (C) encountering vessel branch points upon migration in the microvasculature. **D.** Cells migrating in our tapered microchannel device, which simulates physiological spatial gradients encountered by cells during the metastatic process. The width of the larger channel is 15 µm.

In many instances during the metastatic process, cancer cells encounter spatial gradients. Examples include cells navigating through small pores in the extracellular matrix (ECM) during invasion through the stroma, intra- and extravasation across tight junctions of the endothelium, and migration through the microvasculature especially in vessel branch points [1], [3], [10], [11] – all illustrated in Fig. 1. Essentially, in many scenarios in which a cell interacts with an interface where its degrees of freedom of motion or effective mobility are changed, the local microenvironment exhibits a spatial gradient. Understanding the mechanical responsivity of a cell when encountering spatial gradients, particularly in the context of squeezing into tight spaces during invasion, can elucidate phenomenological attributes associated with metastatic cells.

A number of recent studies have used microfluidic and micropatterning techniques to simulate microenvironments that may be relevant to cancer cell migration. Asymmetric patterns generated on 2D substrates via etching or microcontact printing have been shown to direct cell motion [12]–[14]. Studies using long and straight microchannels with small cross-sectional areas comparable to the cell size, which simulate confined paths in tissues, the microvasculature, and lymphatic vessels [10], have shown that in such environments, cells are able to move unidirectionally with unusually high persistence as compared to 2D studies on flat substrates with no confining boundaries [15], [16]. The physiological microenvironment, however, is diverse and non-uniform. Therefore, straight microchannels, a zeroth order environment (i.e. no perturbations in the direction of cell migration), provide limited means of extracting information about a cell's responsivity. By introducing small perturbations, higher order effects can be examined that may allow one to better understand how individual cells respond to a perturbation to its steady-state.

To accomplish this, here we have developed and conducted cell migration experiments in spatially tapered microchannels with cross-sectional areas comparable to the cell size. This provides a good model for cell navigation through physical constraints and spatial gradients, which are important during metastasis. Typical experiments (Fig. 1 d) for weakly and strongly metastatic cells in these environments are shown in videos S1 and S2, respectively. We demonstrate and compare the mechanical responsivity of three cell types: 1) bovine aortic endothelial cells (BAECs), which are a primary cell culture used here to provide basic insights toward mechanical and migratory behavior of adherent cells in tapered microchannels, 2) MCF-10A, a non-transformed human mammary epithelial cell line used here to represent non-metastatic cells, and 3) MDA-MB-231, a highly metastatic human cell line derived from metastatic breast carcinoma used in this study to model highly metastatic cells.

To date, most experiments involving engineered microenvironments and cell mechanics have been considered only in the steady-state. For instance, chemotactic responses, migration through straight confinement channels, and many other studies of cell migration, polarization, and morphology have only been characterized by average and steady-state velocities, directional persistence, and other ensemble averaged mechanical properties [15]–[18]. Cell behavior, however, is governed by both spatially and temporally varying molecular signals and feedback [19]–[23]. These transient dynamics, such as the activation of intracellular processes in response to external mechanical or chemotactic stimuli, have not been considered in great detail. In this study, we investigate the transient cell dynamics caused by spatial, physical gradients.

## Results

### Heterogeneity and Statistical Behavior

**Figure 2:**
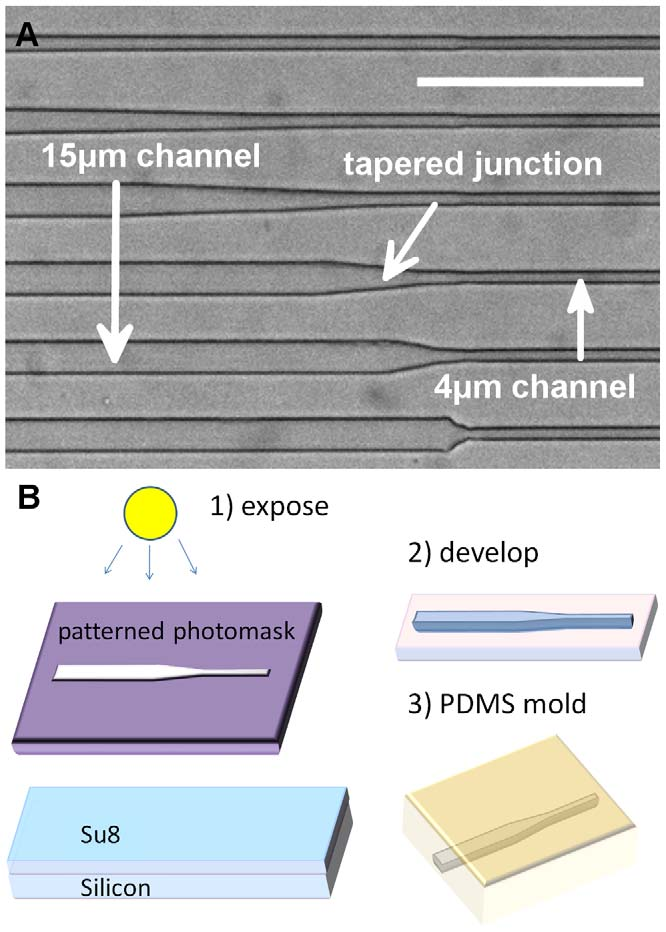
Design patterns and device fabrication process. **A.** Image of actual device tapered microchannels from brightfield microscopy. Larger channels (15 µm×10 µm) are connected to smaller channels (4 µm×10 µm) via a tapered junction. The tapering angles are 1, 2, 3, 7, 15, and 40 degrees from low to high. The first three junctions are considered “low gradients” and the last three are considered “high gradients.” Scale bar 100 µm. **B.** Schematic of fabrication procedure. Standard contact photolithography is used to pattern SU8 masters which are then used in PDMS soft lithography to generate microchannels.

To investigate the migratory response of cells to physical spatial gradients, we designed an array of PDMS microchannels bonded to a glass substrate. The device design and fabrication procedure are shown in Fig. 2. Each channel consists of a tapered junction of variable spatial gradient that connects a large (cross-sectional area: 15 µm×10 µm) channel with a small (4 µm×10 µm) channel. Six different gradients are incorporated, and for the studies here they are categorized as either “high” (tapering angle larger than 7 degrees) or “low” (tapering angle smaller than 3 degrees) gradients (see Fig. 2 caption for more details). Cells migrate unidirectionally in the large channel towards the small channel and their behavior in the tapered region is observed via timelapse microscopy (approximately 24 hrs per experiment) and analyzed. See methods section for more details on the fabrication of the microfluidics.

**Figure 3:**
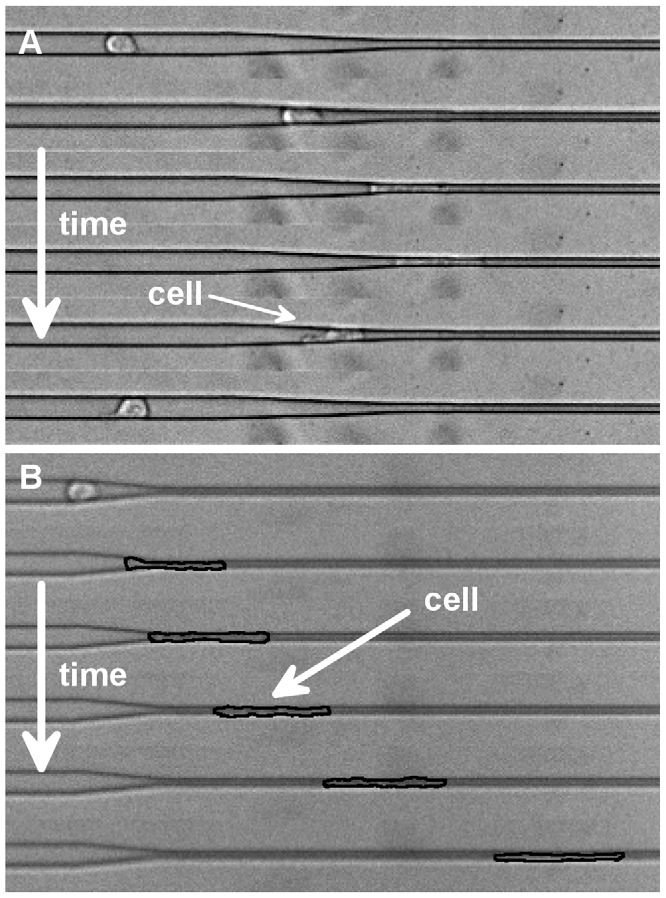
Heterogeneous cell behavior in tapered junctions. **A–B.** Timelapse montage of a cell (MCF-10A) (A) turning back once reaching the tapered region (each frame is 0.96 hrs) and (B) permeating into the smaller channel (each frame is 2.5 hrs). Width of larger channel is 15 µm. See supplementary materials for timelapse videos of all 3 cell types studied.

We characterize the cell as a two-state system where each state corresponds to its polarization, which is determined here based on the direction of cell migration. Since the cell is confined to migrate in 1D, only two polarizations exist, forward and backward. We measure the probability of occurrence of each state upon a cell's interaction with the tapered geometry. Specifically, the two states are determined as: 1) a cell penetrating through the tapered junction and permeating into the smaller channel (*i.e.* the entire cell body is inside the smaller channel), and 2) a cell turning around (repolarizing) once reaching the tapered region and migrating in the backwards direction. Sample experiments demonstrating both states are shown in Fig. 3, and Videos S3, S4, S5, S6 show timelapse movies of various cell types migrating in the devices and exhibiting various behaviors. All cells considered are initially migrating in the direction pointing from the larger channel to the smaller channel. To account for random repolarizations due to distance traveled and the different lengths of tapered junctions of different spatial gradients, a fixed interaction length (250 µm between tapered region and start of small channel) is considered for each cell. All and only cells entering this region are considered, so random repolarizations due to distance traveled are weighted equally in all junctions. Furthermore, cells that die or have not made a conclusive decision by the end of each timelapse experiment are ignored. Cells interacting with other cells are also ignored.

First, our results demonstrate the non-trivial existence of these two states, as both have been observed with appreciable frequency. We have identified two distinct migratory phenotypes, permeating cells and repolarizing cells. Here, phenotype refers to any observable characteristic or behavior of the cell. The occurrence of these two states enables us to quantify migratory invasiveness both in the same cell population and across different cell types with a simple binary analysis. We characterize these events by their probability of occurrence and show that there is a significant dependence of this property on both the spatial gradient of the tapered junction and the cell type.

**Figure 4:**
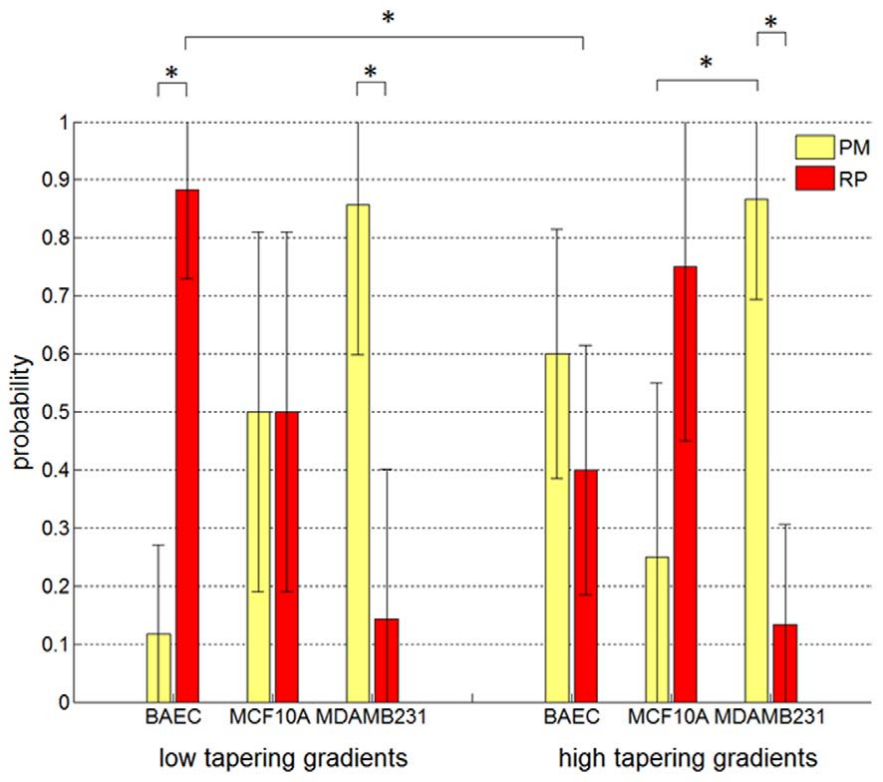
Cell behavior statistics at the tapered junction. Data plots showing the probability of cell permeation (PM, yellow) and repolarization (RP, red) for low and high gradient tapers for three different cell types. Error bars represent 95% confidence intervals calculated from the standard deviation of Bernoulli experiments. * denotes statistical difference, p-value<.05, between data at the nodes of each line.

Fig. 4 shows the response of different cell types to the spatial gradient of the tapered junction. For BAECs, the probability of permeation (into the small channel once reaching the tapered region from the large channel) is greater for higher spatial gradients (60%, n = 20) than for lower gradients (12%, n = 17) (p<.05). In other words, more gradual transitions tend to induce cells to repolarize more often. Furthermore, for the subset of cells that experience this more gradual transition, the probability of repolarization (88%) is statistically higher than permeation (12%) (p<.05). For MCF-10A's, the probability of permeation is 50% (n = 10) for low gradients and 25% (n = 8) for high gradients. For MDA-MB-231's, the probability of permeation is 86% (n = 7) for low gradients and 87% (n = 15) for high gradients.

The response of highly metastatic MDA-MB-231 cells shows several distinguishing features. First, the probability of permeation for both low and high gradients is statistically greater than the probability of turning around (p<.05). For low gradient tapers, this result is opposite to that of BAECs. Next, in comparison with non-metastatic breast epithelial cells (MCF-10A's), MDA-MB-231 cells exhibit a statistically higher (p<.05) probability of permeation for high gradient tapers. These differences, particularly the latter case, can potentially be distinguishing factors between highly metastatic cells and non-metastatic cells. A higher probability of permeation in a spatially tapered and highly confining microenvironment for a particular cell type may indicate greater invasiveness in the context of cancer metastasis.

It has been shown previously that small channels which force cells to deform significantly in order to enter have a much lower probability of cell permeation upon contact [16]. Our results, particularly for MDA-MB-231's, show that there is a substantial permeative population into the smaller channel despite such highly constrictive spatial domains. This may imply that once a cell has entered into a mode of 1D unidirectional migration, its permeative and invasive capabilities are enhanced, at least in the direction of motion. Physiologically this may suggest that there is a feedback mechanism that once a metastatic cancer cell has entered into a defined track in the extracellular matrix or microvasculature, it gains increased aggressiveness during invasion into more confining spaces.

### Cell Transition Dynamics and Signaling Feedback on the Single Cell Level

The tapered channel assay presented above can also be used as a label-free method of quantitatively characterizing signaling feedback on the single cell level by analyzing the mechanical responsivity of cells and profiling cell migration transition dynamics. Responsivity is the factor that maps an external input to an output of interest. Here, the input is the transformation of space and the output is the induced cell migration dynamics. Cell dynamics involve intracellular signaling which entails feedback loops to ensure a robust and rapid cell response. Feedback (whether electrical, mechanical, or biological) can often manifest mathematically as an exponential (sigmoid) curve [20], [21]. Therefore, we fit the velocity profile of cells migrating in the spatially tapered region into sigmoid curves and derive characteristic transient constants. We note the sequential activation of two feedback loops (one negative and one positive). The model we used for curve fitting is:
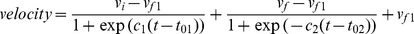
where *v_i_* is the initial steady-state velocity, *v_f_* is the final steady-state velocity, *1/c_1_* is the time constant of the first sigmoid, *1/c_2_* is the time constant of the second sigmoid, *t_01_* is the time for the mid-point of the first sigmoid, *t_02_* is the time for the mid-point of the second sigmoid, and *v_f1_* is the final steady-state velocity if the second sigmoid is not present. By analyzing the temporal evolution of the cell's velocity, we can extract several key parameters of the transition process – 1) the time constants of the sigmoid curves (the net signaling feedback loops) and 2) the temporal delay between the activation of the two net signaling processes (*t_02_*−*t_01_*).

The first process is a negative feedback loop that diminishes the speed of the cell as it encounters additional spatial constraints (*i.e.* the spatial taper). The second process is a positive feedback loop that accelerates the cell to a steady-state velocity in the direction it has chosen to pursue after encountering the spatial gradient. The delay in the activation of these two signaling processes is likely time used to reorganize the cell's cytoskeletal network for permeation into a more confining channel or repolarization for migration in a different direction.

**Figure 5:**
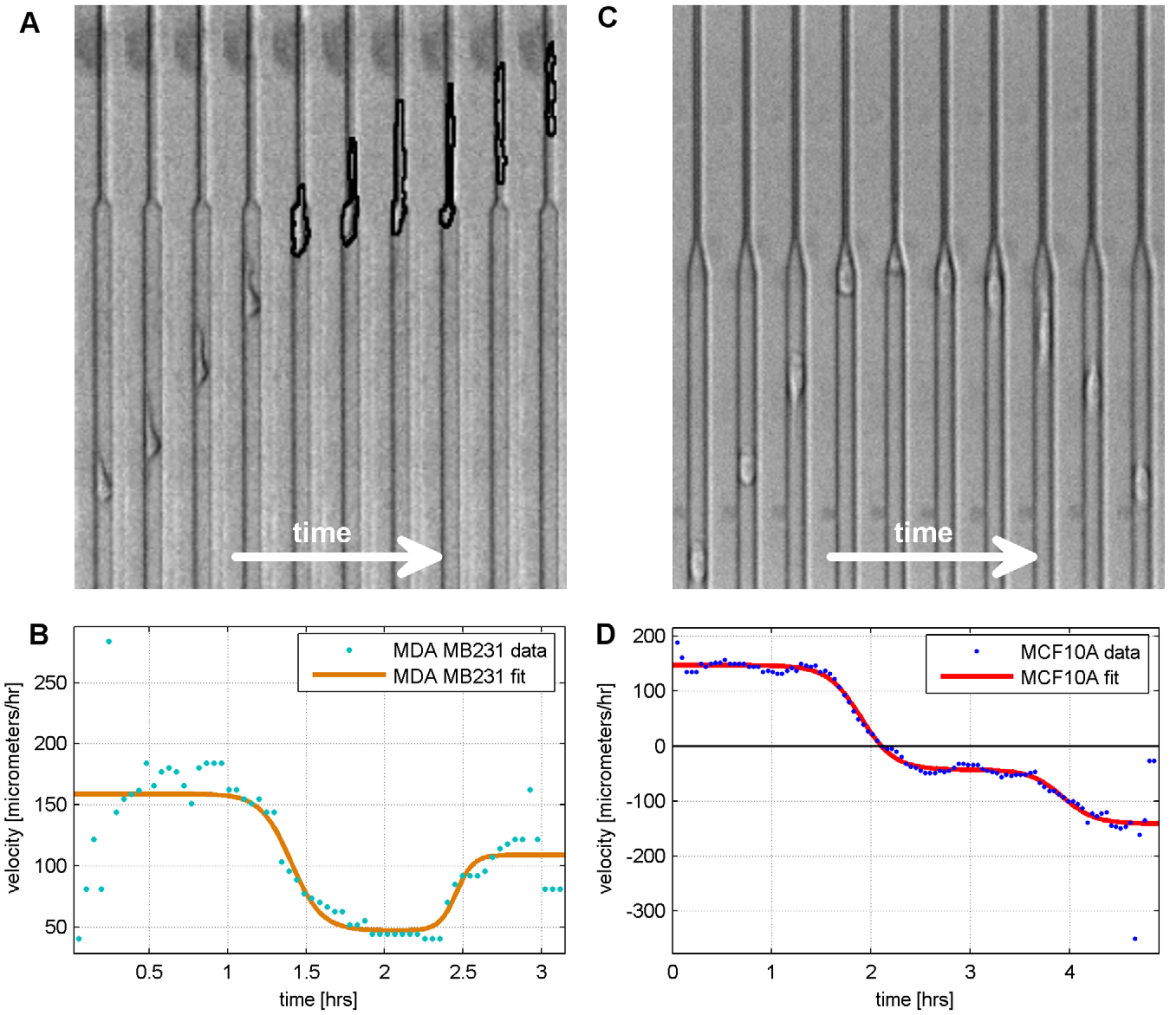
Migration dynamics of repolarizing and permeating cells. **A–B.** Timelapse image stack (A) juxtaposed on top of the data and sigmoid curve fit of the velocity profile on the same time interval (B) of a permeating MDA-MB-231 cell during transition in the tapered junction. The time constants for the first and second sigmoidal curves are approximately 6 and 3 minutes, respectively, and the delay constant is 1 hour. R^2^ of the fit is 0.7. **C–D.** Timelapse image stack (C) juxtaposed on top of the data and sigmoid curve fit of the velocity profile on the same time interval (D) of a repolarizing MCF-10A cell during transition in the tapered junction. The time constants for the first and second sigmoidal curves are both approximately 10 minutes, and the delay constant is 2 hours. R^2^ of the fit is 0.94.

Two time constants and a delay constant provide suitable curve fits for the velocity profile of cells undergoing this transition. For example, as shown in Fig. 5a for a permeating cell, the two time constants are 6 and 3 minutes, respectively, and the delay constant is 1 hour, and as shown in Fig. 5b for a repolarizing cell, the two time constants are both approximately 10 minutes and the delay constant is 2 hours. Time and delay constants for different cells can vary (from minutes to hours) indicating potentially diversity in signaling pathways at play and the cytoskeletal complex of individual cells. This illustrates the importance of considering single cell dynamics rather than ensemble averages, particularly for the analysis of cancer cell mechanics since metastatic potential may be dictated by heterogeneous subpopulations displaying more invasive characteristics [7]–[9]. This method presents a way of measuring the signaling of a net biological process which may be more meaningful than the expression of individual signaling molecules that may contribute to a multitude of pathways and phenotypes.

## Discussion

In this study, we have investigated the migratory behavior of different cell types in response to physical spatial gradients. We focused on the transition region connecting a larger channel to a smaller channel and demonstrated the effect of varying the spatial gradient of the junction on cell responsivity. We also showed that the highly metastatic cells used here (MDA-MB-231's) have a statistically higher permeative nature into smaller regions than non-metastatic cells (MCF-10A's), at least when the spatial gradient is high.

Previous work that used highly confining environments to study cell migration and adhesion dynamics has primarily involved straight and symmetric microchannel structures. For example, Jacobelli *et al.* [24] demonstrated that myosin IIA regulates the crawling mode of T-cell migration by analyzing the “walking” and “sliding” adhesion dynamics of T-cells when migrating in straight confinement microchannels. Hawkins *et al.* [25] developed a mathematical model that addresses spontaneous motion in narrow channels based on actin polymerization within a model cell. Furthermore, Irmia and Toner [15] demonstrated that mechanical confinement can induce spontaneous unidirectional migration in cells, and migration rates are affected by microtubule-disrupting drugs such as Taxol and Nocodazole. These studies clearly showed the rich information about cell mechanics and motility that can be extracted by imposing physical constraints in the cells' local microenvironment. However, the data from these previous experiments were based on spontaneous cell reactions in a static environment with no perturbative features for stimulating cell responsivity. Very little information can be extracted about cell behavior at barriers and interfaces, which is especially important when considering metastasis, during which cancer cells are often transitioning across impeding junctions and into new environments. One such interface is the increase in physical constraint (as illustrated in Fig. 1), which our assay simulates. By introducing a spatially tapering region into microchannels, cells are stimulated at the interface and responses are induced. Therefore, stimulated dynamics rather than steady-state or spontaneous reactions can be studied.

Additionally, we elucidated the existence of behavioral differences within a common cell type in response to a tapered microgeometry; all cell types used here exhibited both permeating and repolarizing subpopulations. The existence of these two states demonstrates phenotypic heterogeneity in mechanical invasiveness among a common population of cells. Whether this heterogeneity is static or dynamic (*i.e.* whether the same cells always exhibit the same phenotype or this phenotype fluctuates in time for all cells) and the implications of either are currently not known and further studies are required. Heterogeneous subpopulations in tumors have been shown [26], [27], but the contributions to metastatic potential are not well understood [5], [7], [8]. Our technique presents a way of probing this heterogeneity based on mechanical properties on a single cell level.

Furthermore, migration dynamics under transition phases can provide insights into the mechanical responsivity of cells that can ultimately be mapped to intracellular signaling feedback mechanisms. For instance, one of the key contributors to cell locomotion is the actin machinery, where the polymerization and depolymerization of actin filaments provide force that drives cells in the direction of motion [28], [29]. The velocity of cells then should be approximately proportional to the number of actively contributing actin filaments, and the velocity profile measured in this study should therefore be representative of actin signaling dynamics (*i.e.* the concentration profile of actin in the polarized edge of the cell, with negative values indicating that the polarization has changed directions).

Finally, our data indicate that metastatic MDA-MB-231 cells exhibit a more invasive phenotype (greater motility through the high gradient channels) than non-metastatic MCF-10A and BAECs. Because metastasis is a highly physical process that involves cell migration and deformation, our microfabricated system may have uncovered a novel mechanism by which metastatic cells enter narrow capillary beds of organs – cells may move through capillaries through active migration rather than simply passive flow transport. In our system, high and low gradient tapers may simulate vessel branch points and continuation along the main branch, respectively. Overall, by introducing additional parameters, *e.g.* variable geometric constraints, in engineered microenvironments, more information can be deduced about cell-environment interactions, such as mechanical triggers for cell repolarization and stability and persistence of cell polarization when perturbed externally. Investigation of the migratory response of cells to spatial constrictions could be valuable in elucidating other mechanical markers of metastasis.

As with most *in vitro* experimental systems, there are important caveats to address. Two important properties are the compliance of the materials used and the dimensionality of the system compared with physiological environments. The boundaries of our microchannels are glass, which is effectively purely rigid, and PDMS (10∶1 ratio of silicone elastomer to curing agent), which has an elastic modulus of around 10^3^ kPa [30], [31]. Typical physiological surfaces that cells adhere to are soft and viscoelastic tissues comprising of the extracellular matrix and other cells (with elastic moduli between 10–10000 Pa) [32]–[35], which can be deformable under cellular forces [30], [32], [36], [37]. Strong connective tissue and blood vessel walls can have elastic moduli on the order of 1 MPa [31], [33]. The complexity of the physiological environment, with such properties as non-uniform pore sizes and varying viscoelasticity in addition to dynamically regulated chemical signaling and proteolysis [35], [38]–[42], makes it difficult to quantitatively analyze the fundamental principles of any physical processes. To begin to derive the governing properties of cell migration and invasion, it is important to simplify the experimental domain. With our assay, we are essentially considering a limiting case in which the compliance is low (relative to soft tissues) at the microchannel walls and infinite inside and along the channel. By reducing the width of the channel through physical tapering, we are reducing the “effective compliance” as experienced by the cells. Similarly, the dimensionality of our microchannel system can be considered as either 1-D, since cells are primarily moving along one axis, or pseudo 3-D, since cells can adhere to and interact with the four surrounding walls. Typical experiments that are supposed to mimic more physiological 3-D environments are conducted with cells embedded in extracellular matrix-simulating gels [40], [43], [44]. Fraley *et al.* [45], for example, demonstrated that cell motility in these 3-D environments does not rely significantly on focal adhesion formation and depends on traction between cell protrusions and the surrounding matrix, both of which are different than 2-D motility. While 3-D experiments are excellent in elucidating more physiological mechanisms of cell motility, it is difficult to simulate and modulate interfaces, which as mentioned throughout this paper have important physiological consequences, in 3-D gels. Furthermore, the cell-in-gel model may not be the most accurate with regards to cell dynamics in microcapillaries, where the surrounding matter is the vessel wall and the interior is fluid (*e.g.* Yamauchi *et al.* [10] showed that cell dynamics in micro-vessels are relevant during the metastatic process). One of the main advantages of the confined microchannel approach is the ability to introduce and tune interface geometries. Ultimately, our tapered channel assay enables the quantitative analysis of the ability of a cell to transition from a region with higher degrees of freedom in movement to a region with lower degrees of freedom. Extensions of this assay could incorporate extracellular matrix components and multiple cell types in the channels to simulate more physiological conditions.

## Methods

### Cell Culture

BAECs (VEC Technologies) were maintained at 37°C and 0% CO_2_ in Leibovitz L-15 media supplemented with 10% Fetal Bovine Serum and 1% Pen/Strep. Experimentation was conducted using the same media under the same condition.

MDA-MB-231 cells from the American Type Culture Collection (ATCC, HTB-26) were maintained at 37°C and 5% CO_2_ in DMEM supplemented with 10% Fetal Bovine Serum. Experimentation was conducted in the same condition except with DMEM replaced by L-15 and at 0% CO_2_.

MCF-10A cells from the ATCC (CRL-10317) were maintained at 37°C and 5% CO_2_ in DMEM/F12 supplemented with 5% Horse serum, 0.5 µg/ml Hydrocortisone, 20 ng/ml hEGF, 10 µg/ml Insulin, 100 ng/ml Cholera toxin, 100 units/ml Penicillin, and 100 µg/ml Streptomycin. Experimentation was conducted in the same condition except with the addition of 10 mM HEPES buffer and at 0% CO_2_. During experiments, the pH of cell culture media was monitored periodically by observing the color of the media due to the phenol red dye. No significant changes were seen. Furthermore, fresh media with the addition of 10 mM HEPES buffer for pH stabilization were replenished every 24 hours.

Note: The media used for each cell type are based on the ATCC (American Tissue Culture Collection) or National Institutes of Health Physical-Sciences and Oncology Center specifications, also delineated by Debnath *et al.* [46] and Guise *et al.* [47].

### Microchannel Fabrication

As shown in Fig. 2b, standard contact photolithography is used to generate an SU8 (MicroChem, Newton, MA) on silicon master that is used to create PDMS (10∶1 silicone elastomer to curing agent ratio) (Dow Corning, Midland, MI) molded microchannels, which are bonded to a glass slide. In the designed pattern, as shown in Fig. 2a, a tapered junction of variable spatial gradient connects a large (cross-sectional area: 15 µm×10 µm) channel with a small (4 µm×10 µm) channel.

### Cell Loading and Preparation for Experiments

Two fluidic injection ports are incorporated into the microchannel device – one on the side of the larger channels (inlet) and one on the side of the smaller channels (outlet). Cells are loaded into the inlet and allowed to proliferate and migrate into the larger channels. During experiments, devices are placed on top of a heating stage maintained at 37°C.

### Cell Migration Trajectory and Velocity Tracing

Timelapse microscopy conducted on an inverted microscope with a 10× objective, with a temporal resolution of 2.88 min/frame, was used to record cell migration in microchannels. The center of mass of cells was tracked manually through image stacks using ImageJ, and velocities were calculated by linear approximation with adjacent frames. Each velocity data point was then averaged with the neighboring 10 points for smoothening and noise filtering.

### Statistical Analysis of Cell Permeation Vs. Repolarization

Since we are considering a binary system and assuming the behavior of each cell represented by the data can be considered as an independent event, the statistics should follow the Bernoulli distribution. The statistical variance *v* of the cell behavior is then *pq*, where *p* and *q* are the probabilities of cell permeation and repolarization, respectively. By the central limit theorem [48] for a sample of size *n*, the error of estimating *p* (and *q*) from our experimentally acquired value of *p_e_* (and *q_e_*) should follow a normal distribution. Mathematically:
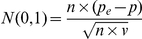
where N(0,1) is notation for the standard normal distribution. To calculate confidence intervals:
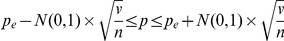
and N(0,1) is 1.96 for 95% confidence and *v* is approximated by our experimental values as *p_e_q_e_*. For further details see [48].

## Supporting Information

Video S1 Sample high-throughput (multichannel) experiment of MCF-10A cells migrating in tapered microchannel device. The frame rate is 10000 times faster than real-time (every second in the video corresponds to 2.78 hours). The widths of the larger and smaller channels are 15 and 4 µms, respectively.
(AVI)

Video S2 Sample high-throughput (multichannel) experiment of MDA-MB-231 cells migrating in tapered microchannel device. The frame rate is 10000 times faster than real-time (every second in the video corresponds to 2.78 hours). The widths of the larger and smaller channels are 15 and 4 µms, respectively.
(AVI)

Video S3 BAEC permeating through the tapered microchannel. The frame rate is 10000 times faster than real-time (every second in the video corresponds to 2.78 hours). The widths of the larger and smaller channels are 15 and 4 µms, respectively.
(AVI)

Video S4 BAEC turning around (repolarizing) once reaching the tapered region. The frame rate is 10000 times faster than real-time (every second in the video corresponds to 2.78 hours). The widths of the larger and smaller channels are 15 and 4 µms, respectively.
(AVI)

Video S5 MCF-10A cell permeating through the tapered microchannel. The frame rate is 10000 times faster than real-time (every second in the video corresponds to 2.78 hours). The widths of the larger and smaller channels are 15 and 4 µms, respectively.
(AVI)

Video S6 MCF-10A cell turning around (repolarizing) once reaching the tapered region. The frame rate is 10000 times faster than real-time (every second in the video corresponds to 2.78 hours). The widths of the larger and smaller channels are 15 and 4 µms, respectively.
(AVI)
